# The Effect of the Menstrual Cycle and Oral Contraceptive Cycle on Muscle Performance and Perceptual Measures

**DOI:** 10.3390/ijerph182010565

**Published:** 2021-10-09

**Authors:** Belinda M. Thompson, Kaitlyn B. Drover, Rhiannon J. Stellmaker, Dean V. Sculley, Xanne A. K. Janse de Jonge

**Affiliations:** 1 Faculty of Medicine, Health and Human Sciences, Macquarie University, North Ryde, NSW 2109, Australia; 2 School of Environmental and Life Sciences, The University of Newcastle, Ourimbah, NSW 2258, Australia; kaitlyn@i-fitness.com.au (K.B.D.); Rhiannon.Stellmaker@uon.edu.au (R.J.S.); x.jansedejonge@outlook.com (X.A.K.J.d.J.); 3 School of Biomedical Sciences and Pharmacy, The University of Newcastle, Ourimbah, NSW 2258, Australia; dean.sculley@newcastle.edu.au

**Keywords:** estrogen, progesterone, menstrual cycle, monophasic oral contraceptives, androgenicity, muscle strength, muscle power, muscle soreness

## Abstract

Most reproductive-aged women are exposed to fluctuating female steroid hormones due to the menstrual cycle or oral contraceptive use. This study investigated the potential effect of the menstrual cycle and combined monophasic oral contraceptive cycle on various aspects of muscle performance. Thirty active females (12 with a natural menstrual cycle, 10 taking a high-androgenicity oral contraceptive and 8 taking a low-androgenicity oral contraceptive), aged 18 to 30 years, were tested three times throughout one menstrual or oral contraceptive cycle. Counter-movement jumps, bilateral hop jumps, handgrip strength, isometric knee extensor strength and isokinetic knee flexion and extension were assessed. Perceptual ratings of fatigue, muscle soreness, pain and mood were recorded. Most variables showed no significant changes over the menstrual or oral contraceptive cycle. However, for the menstrual cycle group, isokinetic knee flexion at 240° s^−1^, and time of flight in bilateral hopping and counter movement jumps showed better results during the mid-luteal phase compared with the late follicular phase. For the high-androgenicity oral contraceptive group, isokinetic knee flexion at 240° s^−1^ was significantly higher in the late hormone phase compared with the early hormone phase. For the low-androgenicity oral contraceptive group, time of flight for the counter-movement jumps was lower in the late hormone phase compared with the early hormone phase. The findings indicate that faster and explosive aspects of muscle performance may be influenced by endogenous and exogenous female hormones.

## 1. Introduction

An important consideration for female athletes is the potential influence of fluctuating levels of endogenous ovarian hormones throughout the menstrual cycle (MC) on performance. In addition, the influence of exogenous hormones from various contraceptive methods should also be considered. Oral contraceptives (OC) suppress the natural fluctuations of estrogen and progesterone through the administration of exogenous hormones. The combined monophasic OC is the most used; however, the chemical formulations vary considerably among the different types. The contemporary combined OC contain an estrogen component paired with one of eight progestins [1]. The progestin agents vary in terms of their potency, androgenicity and interactions with estrogens [2]. Androgenicity denotes the progestin’s ability to produce masculine characteristics and may have implications for muscle strength and function [3]. Newer progestins often reduce the androgenic effects of OC, whereas older progestins derived from testosterone have an increased androgenic effect [4].

The influence of female sex hormones on muscle strength and function, as well as adaptations to resistance training, is not well understood [5]. Hormone replacement therapy research in post-menopausal women has supported the role of estrogen in the maintenance of muscle strength [6]. However, research on the influence of the natural MC on acute performance in assessment of muscle strength and function has provided conflicting results. Several studies have reported significant differences in the performance of muscle strength and function over the MC [7,8,9,10,11,12,13], while others have not observed any differences [14,15,16,17,18].

The limited research on the potential effects of OC on muscle strength and function generally reports no differences in performance over the OC cycle [8,9,19]. In contrast, Rechichi and Dawson (2009) reported a significantly greater reactive strength from drop jumps during the hormone pill phase than during the non-active pill phase [20]. However, Rechichi and Dawson did not consider potential differences in the androgenicity of the OC. It has been proposed that OC containing progestins with higher androgenicity will have greater benefits for muscle function and performance than those with progestins of low androgenicity [21]. To our knowledge, only one study has investigated the potential differences in muscle performance between high and low androgenicity levels of the monophasic OC pill. Burrows and Peters (2007) observed no differences in peak isokinetic knee extension and flexion torque between participants taking a low-androgenicity OC compared with those taking a high-androgenicity type of OC [21]. However, this study only focused on slow isokinetic strength, so it is not known how the androgenicity of OC may affect other measures of muscle performance. In addition, the significant increase in reactive strength in the active hormone pill phase compared to the non-active pill phase reported by Rechichi and Dawson (2009) suggests that further research should include the fast and explosive aspects of muscle performance [20].

The cyclic fluctuations in female hormones have also been associated with a variety of emotional changes, such as depression, irritability, or tension [22]. Early research has shown a relationship between the MC phase and variations in mood [23]. OC use appears to decrease the cyclic changes in psychological variables with females taking OC reporting fewer changes in mood over the cycle [24]. Research has also shown a lower pain threshold in the early follicular phase compared to the late follicular and luteal phases [25]. These factors have the potential to influence performance over the cycle; however, most studies examining muscle function over the MC or OC cycle have not considered these variables.

To address the conflicting findings and gaps in the research to date, this study aims to evaluate the potential effect of the MC and the monophasic OC cycle with both high and low androgenicity on various aspects of muscle performance, including fast and explosive measures, in combination with perceptual measures.

## 2. Materials and Methods

### 2.1. Protocol

All participants in the study provided informed consent and visited the laboratory on five occasions. The first two sessions were familiarisation sessions, performed one week apart. The remaining three sessions were identical experimental trials separated by at least a week. The MC group was tested within the first four days of menstruation (early follicular phase), within 24 h of a positive urinary luteinizing hormone (LH) test (late follicular phase), and 7–9 days after the positive LH test (mid-luteal phase) [26]. The two OC groups were tested between days 4 and 7 of non-active pill consumption (non-active pill phase), between 4 and 7 days of active pill consumption (early hormone phase), and between 17 and 21 days of active pill consumption (late hormone phase) [27]. The MC or OC phase for the first testing session was randomly allocated, to minimise any learning effect. All testing sessions for each participant were scheduled for the same time of day. Participants were asked to be hydrated and to avoid caffeine, alcohol and exhaustive exercise or resistance training of the lower limbs in the 48 h prior to each testing session. At the first session, participants filled out a physical activity log for the past week, which they replicated prior to each of the testing sessions. The working guide for standards of practice for research in women was followed in this study [28].

### 2.2. Participants

Thirty active females aged between 18 and 30 years volunteered to participate in this study. Participants in the MC group (*n* = 12) had not taken any hormonal contraception for at least six months prior to testing and had a regular MC (21 to 35 days). The high OC group (*n* = 10) had taken a monophasic OC with high androgenicity, while the low OC group (*n* = 8) had taken a monophasic OC with low androgenicity for at least six months. Androgenicity of the OC was determined according to Greer et al. (2005) [29]. All procedures were approved by the institutional Human Ethics Committee.

### 2.3. Perceptual Measures

At the start of each testing session, participants completed an abbreviated version of Profile of Mood States (POMS) [30]. The sum of the fatigue-item scores was subtracted from the sum of the vigour-item scores, which resulted in the energy index measure [30]. Participants also completed the visual analogue scales (VAS) for mood, physical fatigue, and muscle soreness.

The final perceptual measure was the pain response to a standardised electrical stimulation. Adhesive electrodes (Dura-stick, Chattanooga, DJO Global Inc., UK) were placed anteriorly on the participant’s dominant leg, 3cm above the patella, and proximally on the thigh. A high-voltage stimulator (DS7A, Digitimer Ltd., UK) was used to deliver a series of percutaneous electrical pulses via the electrodes. The current was increased in 50 mA steps, and when the involuntary force created by the electrical stimuli reached a plateau, the current of the third stimulus in the plateau was defined as the supra-maximal response [16]. This level was recorded and used for all subsequent tests. Four separate pulses were then delivered at this level, with five seconds rest between each pulse. The participants were asked to rate the level of pain created by the supra-maximal electrical stimuli on a VAS.

### 2.4. Muscle Function Measures

The participants performed a 10-minute warm-up on a cycle ergometer (Ergomedic 828 E, Monark, Sweden) at 50 Watts with a cadence of 50 rpm at each session.

Jump testing was performed using a portable force plate (Quattro Jump, Kistler, Switzerland). Participants performed ten maximal continuous bilateral hops. Participants jumped as high as possible, with minimal bending of the knees, minimal contact time and with the hands placed on the hips. Average power (Pavg), contact time (Tcont), flight time (Tflight), and the ratio of flight time over contact time were all recorded. The average of the three best consecutive hops was used for analysis. Following a one-minute rest, the participants performed five repeated counter-movement jumps (CMJ) with the hands placed on the hips. Average power (Pavg) and flight time (Tflight) were recorded and the maximum value was used for analysis.

Handgrip strength was measured using a hand dynamometer (Dynamo Meter, TTM, Tokyo). Participants stood with the arm beside their body with the elbow extended. Three trials were performed, with the best score for each hand used for analysis.

An isokinetic dynamometer (Humac Norm, Computer Sports Medicine Inc, USA) was used to measure isometric quadriceps strength and isokinetic knee extension and flexion torque of the dominant leg. Participants were secured to the chair by hip, shoulder, and thigh straps and held their arms crossed over their chest during the tests. Torque signals were collected onto a data acquisition system (MP150, Biopac Systems Inc., USA) at a sampling rate of 2500 Hz. AcqKnowledge software (version 4.2, Biopac Systems Inc., USA) was used to display real-time visual feedback of torque signals on a computer monitor and to save data for later analysis.

For the isometric quadriceps strength test, the knee joint angle was set at 60° from horizontal. Participants performed five maximum voluntary contractions (MVC), preceded by a 30 s rest period. To check for neural activation during the strength measurements, a supra-maximal twitch was superimposed on each MVC. After a one-minute rest, participants performed five consecutive knee flexions and extensions at a velocity of 60°·s^−1^ and 240°·s^−1^ with a one-minute rest between the different speed tests. The peak force or torque recorded in each session was taken as the MVC. Verbal encouragement was given during all strength tests.

### 2.5. Menstrual Cycle Phase Verification

Participants in the MC group began using urinary LH test strips (Fertility2Family, Australia) to estimate the day of ovulation from day 8 of their cycle until a positive test was recorded [31]. If a positive test was not received by day 25 of their cycle, testing was postponed to the next cycle. At the start of each testing session, venous blood samples were drawn to later measure serum concentrations of estradiol and progesterone to verify MC phase. A minimum progesterone concentration of 16 nmol·L^−1^ during the mid-luteal phase was required for confirmation of an ovulatory cycle [32]. Any participants in the MC group who did not meet the minimum concentration of progesterone in the luteal phase were excluded from further analysis [31].

Venous blood samples were collected directly into two serum separator tubes (BD Diagnostics Systems, NJ, USA). Whole-blood samples were allowed to clot at room temperature before centrifugation (Allegra X-15R, Beckman Coulter, USA) at 1100 RCF for 10 min. Serum samples were stored frozen at −80°C until analysis for estradiol and progesterone. Hormonal analysis was conducted by a professional pathology laboratory (Pathology North-NSW Health, Hunter-New England, National Association of Testing Authorities, Australia accreditation number 3561). Abbott Architect chemiluminescent microparticle immunoassay (Abbott Laboratory, Illinois, USA) was used to measure estradiol and progesterone concentrations. Coefficients of variation reported by the laboratory were 7.3% for estradiol and 6.5% for progesterone.

### 2.6. Statistical Analysis

Data are presented as means ± standard deviation (SD). The software package SPSS (version 24; SPSS Inc., USA) was used to conduct the statistical analysis and the alpha level was set at *p* ≤ 0.05. All data were tested for normality with the Kolmogorov–Smirnov test. Repeated-measures ANOVA was used to assess potential changes over the three phases of the MC or OC cycle. Based on the significance of the Mauchly’s Test of Sphericity, either the Sphericity assumed or Huynh–Feldt results of the Within-Subject Effects were used. For any significant main effects, further post hoc testing was conducted with Fisher’s Least Significant Difference (LSD). Repeated-measures ANOVA was also performed for the test to identify a potential learning effect. The effect size (Cohen’s d) was calculated to examine the magnitude of change. An effect size of greater than or equal to 0.8 was considered a large effect, 0.5 a moderate effect, 0.2 a small effect and less than 0.2 was considered to be trivial [33]. A mixed-model repeated-measures ANOVA was conducted with the between-subject factor of hormonal status (high OC, low OC and MC). Independent *t*-tests were used for between group comparison of participant characteristics.

## 3. Results

### 3.1. Participant Characteristics

The mean ± SD for age, height and weight was 22.1 ± 3.5 years, 165.5 ± 6.2 cm and 57.2 ± 8.3 kg for the participants in the MC group, 22.5 ± 3.3 years, 161.9 ± 5.5 cm, 59.5 ± 10.3 kg for the participants in the low OC group, and 20.7 ± 2.1 years, 165.2 ± 6.9 cm, 64.9 ± 11.6 kg for the participants in the high OC group. There were no significant differences in age, height or weight between groups. The participants in the MC group had a cycle length of 28.3 ± 1.8 days (range: 25 to32 days). All the participants in the OC groups had been taking a monophasic OC for a minimum of 7 months (range: 7 months to 11 years). The OC formulation taken by the high-androgenicity group contained 30 µg estradiol and 150 µg levonorgestrel. The OC formulations taken by the low-androgenicity group varied, with levels of estradiol ranging between 30 µg and 35 µg, combined with one of three different progestins (norethisterone, desogestrel or cyproterone acetate). Further details on the exact OC formulations are provided in a Supplementary File.

### 3.2. Hormonal Analysis

The results from the hormonal analysis are presented in Table 1 (MC group), Table 2 (high OC group) and Table 3 (low OC group). Two participants from the MC group were excluded from further analysis, as their progesterone levels did not meet the requirements for confirmation of an ovulatory cycle (>16 nmol·L^−1^). For the participants in the OC group, the hormone results confirm that natural fluctuations in endogenous hormones were suppressed.

### 3.3. Between Group Analysis

The mixed model repeated measures ANOVA with hormonal status as between subject factor showed no significant differences in any of the measured parameters between the MC (*n* = 10), high-androgenicity OC (*n* = 10) and low-androgenicity OC (*n* = 8) groups.

### 3.4. Test Order (Learning) Effect

The repeated measures ANOVA conducted for the data in the testing order showed no significant differences between tests for any of the outcome measures.

### 3.5. Perceptual and Muscle Function Measures for the Menstrual Cycle Group

No significant changes were found between the three phases of the MC for any of the perceptual scores, handgrip strength or isometric strength (Table 1). However, Tflight for the bilateral hops did show a significant main effect (*p* = 0.007). Post hoc testing revealed that Tflight for the bilateral hops was significantly lower in the late follicular phase than in the mid-luteal phase, with a moderate effect size (*p* = 0.002, d = 0.72). For the CMJ, Tflight also showed a significant main effect (*p* = 0.034) and post hoc testing revealed that CMJ Tflight was also significantly lower in the late follicular phase than in the mid-luteal phase (*p* = 0.003, d = 0.40). There were no significant differences for most of the isokinetic tests. However, flexion at 240° s^−1^ showed a significant main effect (*p* = 0.027) with post hoc testing revealing that knee flexor strength at 240° s^−1^ was lower in the late follicular phase than in the early follicular phase, with a small effect size (*p* = 0.031, d = 0.38), and lower in the late follicular phase than in the mid-luteal phase, with a small effect size (*p* = 0.033, d = 0.46).

Although not significant, moderate effects were observed when comparing the late follicular phase with the mid-luteal phase for bilateral hops Pavg (d = 0.53). Moderate effects were also observed for knee extension torque at 240° s^−1^ when comparing the early follicular phase with the late follicular phase (d = 0.51) and the late follicular phase with the mid-luteal phase (d = 0.72). All other effects sizes were small to trivial.

### 3.6. Perceptual and Muscle Function Measures for the High Androgenicity OC Group

No significant changes were found between the three phases of the high OC for any of the perceptual scores, handgrip strength, bilateral hops, CMJ, isometric strength and most of the isokinetic strength measures (Table 2). However, knee flexion at 240° s^−1^ showed a significant main effect (*p* = 0.018) and post hoc testing revealed that knee flexion at 240° s^−1^ was significantly higher, with a moderate effect size during the late hormone phase than during the early hormone phase (*p* = 0.024, d = 0.50).

Although not significant, for the perceptual measures, moderate effects were observed for pain when comparing the early hormone phase with the late hormone phase (d = 0.56) and the non-active pill phase with the late hormone phase (d = 0.59). Moderate effects were also observed for mood when comparing the non-active pill phase with the early hormone phase (d = 0.60) and for the POMS energy index when comparing the early hormone phase with the late hormone phase (d = 0.51). All other effect sizes were small to trivial.

### 3.7. Perceptual and Muscle Function Measures for the Low Androgenicity OC Group

No significant changes were found between the three phases of the low OC cycle for any of the perceptual scores, handgrip strength, bilateral hops, isometric strength or isokinetic strength (Table 3). However, Tflight for the CMJ did show a significant main effect (*p* = 0.033) and post hoc testing revealed that Tflight for the CMJ was significantly higher with a small effect size during the early hormone phase than during the late hormone phase (d = 0.36).

Although not significant, for the perceptual measures, moderate to large effects were observed for soreness when comparing the non-active pill phase with the early hormone phase (d = 0.65) and the late hormone phase (d = 1.04). Moderate effects were also observed for the POMS energy index when comparing the non-active pill phase with the early hormone phase (d = 0.73) and when comparing the early hormone phase with the late hormone phase (d = 0.50). Pain also showed moderate effects when comparing the non-active pill phase with the late hormone phase (d = 0.58). For the strength measures, moderate effects were observed for 240° s^−1^ knee extension when comparing the non-active pill phase with the late hormone phase (d = 0.51). All other effect sizes were small to trivial.

## 4. Discussion

The present study included several different measures of muscle performance, ranging from handgrip strength to explosive power in CMJ. For maximal handgrip strength no significant changes were found over the three phases of the MC and OC cycle, which agrees well with recent research on the MC [8,11,16] and OC cycle [8,11]. Similarly, our findings of no significant differences in isometric knee extension strength or knee extension and flexion torque at 60° s^−1^ align well with previous research over the MC [8,16,18] and OC cycle [8,9,19]. Therefore, it appears that maximal isometric strength and torque at low velocities are not influenced by endogenous or exogenous hormonal fluctuations, due to the MC, high-androgenicity OC or low-androgenicity OC cycle.

To date, most research on muscle function over the MC or OC cycle has focused on isometric strength and/or low-velocity torque, limiting its application to explosive activities and sports. The comprehensive approach of including fast and explosive muscle function measures, as well as perceptual measures, at three distinctly different hormonal time points in the MC, high-OC and low-OC cycle uncovered novel findings in the present study. For the MC group, time of flight for the bilateral hops was significantly lower, with moderate effect sizes, during the late follicular phase than during the mid-luteal phase. Similarly, the time of flight for the CMJ was also lower during the late follicular phase than during the mid-luteal phase, but there was no difference between the early follicular and mid-luteal phases. This is in agreement with Julian et al. (2017), who also reported no significant difference in CMJ performance between their two testing sessions in the early follicular and mid-luteal phase [34]. However, in the current study, the inclusion of testing during the late follicular phase to investigate the potential effect of high estrogen combined with low progesterone uncovered a decrease in jump performance at that time. Furthermore, the present study assessed isokinetic knee flexion and extension torque at the fast velocity of 240° s^−1^, as this measure has been found to be related to explosive jump and sprint performance [35]. The current study showed no change over the MC cycle for knee extension at 240° s^−1^, but, similar to the jumping results knee flexion at 240° s^−1^, showed a reduced performance in the late follicular phase compared with the mid-luteal phase. Gordon et al. (2013) also tested knee extension and flexion at 240° s^−1^ over the MC and reported no significant differences [9]. However, they did not test during the late follicular phase, and therefore the different hormonal environments at the times of testing may explain the conflicting findings. A potential explanation for the decrease in explosive performance during the late follicular phase compared to the mid-luteal phase may be found in changes in other muscle and tendon properties. Research over the MC has demonstrated significant reductions in lower limb musculotendinous stiffness [36] and the muscle stretch reflex [37], and an increase in muscle extensibility [38], joint laxity and risk for injury [39] during ovulation (when estrogen is high, and progesterone is low). Lower limb stiffness is considered important for enhancing jumping and hopping activities, as compliant muscles and tendons reduce the rate of force development and increase electromechanical delay [36]. The changes in musculotendinous stiffness over the MC may, therefore, explain the reduced jump performance and fast isokinetic knee flexion observed in the present study during the late follicular phase, when estrogen is elevated and progesterone remains low. Although the role of progesterone is poorly understood, it has been suggested that progesterone may interfere with estrogen’s effect on the neuromuscular system [20]. Therefore, although estrogen remained high in the mid-luteal phase, progesterone concentration was also high, which may have attenuated the negative influence of estrogen on jump performance.

For the low- and high-androgenicity OC groups performance in the bilateral hops showed no change over the OC cycle and CMJ showed no change over the OC cycle for the high-OC group. For the low-OC group, however, a reduction in CMJ time of flight during the late hormone phase was found compared with the early hormone phase. To our knowledge, only one other study has assessed jump performance over the OC cycle. Rechichi and Dawson (2009) assessed jump performance in 10 participants who were taking a combined monophasic OC during the early non-active phase (days 2-3), the late non-active phase (days 6-7) and during the late hormone phase (days 20-24) [20]. The authors reported no significant differences in CMJ performance over the OC cycle, suggesting that the exogenous estrogen and progestin in the OC do not affect jump performance. However, as the participants were not divided into high- and low-androgenicity groups, it is not known whether androgenicity may have affected the results. It has been suggested that low-androgenicity OC may negatively affect performance. Research has demonstrated increased serum concentrations of ethynyl estradiol with a twofold accumulation from day one of the active pill to day 21 with combined monophasic OC [40,41]. In addition, serum concentrations of progestin reached a steady state concentration after eight [40] and eleven [41] days of hormone pill consumption with a three-fold accumulation. Therefore, the current study planned for the exogenous hormone levels to be different between the early and late hormone phases. It could be that the increased levels of progestin with a low androgenicity in the late hormone phase resulted in a reduction in performance in the low-OC group. However, this conjecture requires further investigation.

Alternatively, our measurement of perceptual data may provide an explanation. The participants completed a VAS for muscle soreness and although there were no significant differences in soreness over the cycle for the low-OC group, a large effect size was observed between the non-active pill and late hormone phase (d = 1.04). As jumping is a whole-body movement, greater soreness throughout the body would likely affect jump performance and may partly explain the decrease in CMJ performance in the late hormone phase in the low-OC group.

For the high-androgenicity OC group, knee flexion torque at 240° s^−1^ was found to be significantly higher in the late hormone phase compared to the early hormone phase (*p* = 0.024). This finding suggests that the exogenous hormones in high-androgenicity OC may have a positive effect on high-velocity strength during the late hormone phase, when exogenous hormone levels are likely to be at the highest levels. To our knowledge, only one other study has tested knee flexion and extension torque at 240° s^−1^ over the OC cycle, and they found no change in their six participants using monophasic OC [9]. As no information was provided regarding the type of monophasic OC used by the participants in the study by Gordon et al. (2013) [9], it is difficult to make comparisons with the high- and low-androgenicity groups in the current study. Our results suggest that the exogenous hormones in the high-androgenicity OC may be beneficial for muscle function at high velocities, but it is difficult to explain why this was only found for knee flexion and not for extension.

Our comparison between the three groups showed no significant differences in acute performance of various measures of muscle function between participants using an OC and those with a natural cycle, which aligns well with previous research [8,19,42]. Similarly, our finding of no significant differences in muscle performance between the high- and low-androgenicity OC groups also agrees with the limited research to date [3]. However, a limitation to the present study is that although the OC formulations were the same for the high-OC group (30 µg estradiol and 150 µg levonorgestrel), the low-OC formulations consisted of varying levels of estradiol (between 30 µg and 35 µg) paired with one of three different progestins (norethisterone, desogestrel or cyproterone acetate). Further research should attempt to have the same formulation of OC within each group.

## 5. Conclusions

This study on muscle performance found that isometric strength and muscle function at slow velocities did not change over the MC, high-androgenicity OC or low-androgenicity OC cycle, which aligns well with the current literature. However, some of the faster and more explosive measures showed significant differences over the MC and OC cycle. For the MC group, isokinetic knee flexion at 240° s^−1^, time of flight in bilateral hopping and time of flight in CMJ showed better results during the mid-luteal phase than during the late follicular phase. These findings suggest that high levels of endogenous estrogen combined with low levels of progesterone may have a negative effect on explosive and high-velocity strength, which may be due to an estrogen-related decrease in musculotendinous stiffness.

For the high-androgenicity OC group, isokinetic knee flexion at 240° s^−1^ was significantly higher in the late hormone phase compared with the early hormone phase. This finding suggests that increased levels of the exogenous hormones in high-androgenicity OC may have a positive influence on high-velocity strength. In contrast, for the low-androgenicity OC group, time of flight for the CMJ was lower in the late hormone phase compared with the early hormone phase, which suggests that increased levels of low androgenicity exogenous hormones may have a negative effect on explosive power. Even though no significant differences were observed over the MC or OC cycle for any of the perceptual measures, muscle soreness had a large effect size for the low-OC group when comparing the non-active pill phase with the late hormone phase, which may partly explain the reduced flight time in CMJ at that time.

The current study is only the second study to compare muscle function characteristics between participants taking an OC with high androgenicity and those taking one with low androgenicity. In agreement with the earlier study, no significant differences between high and low androgenicity were found for any of the measured muscle function characteristics in the current study. Furthermore, the current study showed no differences between the androgenicity groups for the perceptual measures. Unfortunately, both the current study and the earlier work were limited by the low participant numbers in each group. Future research should focus on larger participant numbers with a stricter control of OC type in each group to investigate the potential effect of androgenicity of OC on muscle function and perceptual measures.

The practical implications of the current findings are that, for women who are experiencing a natural MC, it may be beneficial to train for fast and explosive aspects of performance during the luteal phase, when both estrogen and progesterone are high. However, many females and athletes use OC to regulate the timing of their cycle, including skipping the non-active pills to avoid menstruation during competition and times of important training. The outcomes of our research suggest that besides avoiding menstruation, the continuation of the active pill with high androgenicity may benefit explosive and fast movement strength in females and athletes and may potentially result in higher quality training and performance. For females taking a high-androgenicity OC who compete in fast and/or explosive sports, it may be beneficial for performance to ensure that important competitions coincide with the late hormone phase of their OC cycle. Therefore, the manipulation of cycle length (continuation of consumption of the active pills) to align with competition may provide a competitive edge for female athletes in explosive sports. However, further research into the wide and varied aspects surrounding the OC pill with larger participant numbers is needed to enable women to make educated and informed decisions about OC use to control their hormones and cycle around training and competition.

## Figures and Tables

**Table 1:** Hormone concentrations, perceptual and muscle function measures for the Menstrual Cycle group (*n* = 10) over the three phases of the menstrual cycle.

	Early Follicular	Late Follicular	Mid-Luteal	*p*-Value
Estradiol (pmol/L)	**142.6 ± 52.9**	**475.7 ± 275.6**	**577.9 ± 265.2**	0.000 *
Progesterone (nmol/L)	**0.79 ± 0.30**	**6.93 ± 3.95**	**39.87 ± 14.16**	0.000 *
**Perceptual Scores**				
Pain	4.9 ± 2.2	4.3 ± 2.1	4.9 ± 2.3	0.733
Mood	2.6 ± 1.6	2.7 ± 1.8	3.0 ± 1.9	0.872
Soreness	2.1 ± 2.1	2.2 ± 2.2	2.0 ± 2.1	0.969
General Fatigue	3.0 ± 1.7	3.3 ± 1.8	3.4 ± 2.7	0.793
POMS Energy Index	2.9 ± 8.7	4.3 ± 6.2	4.7 ± 10.3	0.778
**Bilateral Hops**				
*Pavg (W/kg)*	26.3 ± 6.0	*25.5 ± 4.1*	*27.8 ± 4.6*	0.079
Tcont (ms)	204.1± 24.0	201.5 ± 25.5	204.7 ± 20.3	0.791
**Tflight (ms)**	**336.6 ± 52.2**	**323.5 ± 35.1 ^#^**	**352.6 ± 45.5**	0.007 *
Ratio (Tflight/Tcont)	1.64 ± 0.36	1.60 ± 0.28	1.68 ± 0.27	0.320
**Counter movement jump**				
Pavg (W/kg)	20.9 ± 3.5	19.9 ± 2.6	20.1 ± 1.7	0.518
**Tflight (ms)**	**427.8 ± 38.3**	**416.0 ± 33.2 ^#^**	**429.8 ± 36.0**	0.034 *
		**Handgrip**		
Right (kg)	35.6 ± 5.8	36.5 ± 4.7	37.1 ± 4.7	0.146
Left (kg)	33.1 ± 5.5	31.8 ± 4.3	32.4 ± 5.4	0.510
**Isometric Strength**				
Knee Extension (Nm)	164.7 ± 36.4	158.8 ± 31.0	166.1 ± 32.8	0.297
**Isokinetic Torque**				
60° s^−1^ Extension (Nm)	113.4 ± 18.6	113.5 ± 18.0	114.0 ± 23.0	0.993
60° s^−1^ Flexion (Nm)	80.4 ± 13.2	81.8 ± 18.9	81.4 ± 14.6	0.904
*240° s^−1^ Extension (Nm)*	*70.0 ± 6.2*	*65.6 ± 10.9*	*72.4 ± 8.0*	0.076
**240° s^−1^ Flexion (Nm)**	**61.8 ± 12.6**	56.7 ± 14.3 ^#,†^	**62.6 ± 11.3**	0.027 *

Data shown as mean ± SD. * **Bold** Significant change over the cycle (*p* < 0.05). ^#^ Post hoc test significantly different from mid luteal. **^†^** Post hoc test significantly different from early follicular. *Italic* Moderate or large effect size between phases. Pavg: average power, Tcont: contact time, Tflight: flight time.

**Table 2:** Hormone concentrations, perceptual and muscle function measures for the high-androgenicity Oral Contraceptive group (*n* = 10) over the three phases of the oral contraceptive cycle.

	Non-Active Pill	Early Hormone	Late Hormone	*p*-Value
Estradiol (pmol/L)	104.9 ± 35.2	87.4 ± 26.6	87.4 ± 26.6	0.051
Progesterone (nmol/L)	0.47 ± 0.3	0.44 ± 0.3	0.46 ± 0.2	0.796
**Perceptual Scores**				
*Pain*	*3.8 ± 1.8*	*3.7 ± 1.7*	*2.7 ± 1.9*	0.221
*Mood*	*2.5 ± 1.0*	*1.9 ± 1.0*	2.4 ± 1.4	0.507
Soreness	1.5 ± 2.0	2.1 ± 1.9	1.6 ± 2.0	0.630
General Fatigue	3.0 ± 1.7	3.3 ± 1.8	3.2 ± 1.9	0.817
*POMS Energy* *Index*	4.6 ± 8.9	*7.6 ± 7.3*	*4.4 ± 5.3*	0.445
**Bilateral Hops**				
Pavg (W/kg)	29.3 ± 6.9	29.1 ± 5.2	30.2 ± 6.5	0.734
Tcont (ms)	199.9 ± 37.4	201.6 ± 39.0	196.1 ± 36.8	0.517
Tflight (ms)	351.4 ± 48.1	359.5 ± 40.4	364.2 ± 45.4	0.413
Ratio (Tflight/Tcont)	1.77 ± 0.36	1.80 ± 0.39	1.88 ± 0.46	0.307
**Counter movement jump**				
Pavg (W/kg)	20.9 ± 3.7	20.4 ± 2.9	21.6 ± 4.5	0.344
Tflight (ms)	422.6 ± 45.9	421.0 ± 47.5	431.6 ± 49.1	0.193
		**Handgrip**		
Right (kg)	34.6 ± 6.2	34.3 ± 5.1	35.4 ± 6.0	0.373
Left (kg)	32.2 ± 5.0	32.5 ± 5.2	33.2 ± 5.7	0.333
**Isometric Strength**				
Knee Extension (Nm)	176.0 ± 41.6	183.2 ± 52.2	184.2 ± 45.4	0.349
**Isokinetic Torque**				
60° s^−1^ Extension (Nm)	134.3 ± 33.6	139.0 ± 36.1	133.4 ± 31.5	0.385
60° s^−1^ Flexion (Nm)	93.1 ± 22.3	95.2 ± 20.3	96.1 ± 18.1	0.524
240° s^−1^ Extension (Nm)	67.8 ± 19.1	74.7 ± 20.7	76.0 ± 20.8	0.213
***240°*** ***s^−1^ Flexion (Nm)***	**65.1 ± 14.3**	***62.8 ± 11.0 ^#^***	***69.8 ± 16.9***	0.018 *

Data shown as mean ± SD. * **Bold** Significant change over the cycle (*p* < 0.05). ^#^ Post hoc test significantly different from late hormone phase. *Italic* Moderate or large effect size between phases. Pavg: average power, Tcont: contact time, Tflight: flight time.

**Table 3:** Hormone concentrations, perceptual and muscle function measures for the low-androgenicity Oral Contraceptive group (*n* = 8) over the three phases of the oral contraceptive cycle.

	Non-Active Pill	Early Hormone	Late Hormone	*p*-Value
Estradiol (pmol/L)	95.0 ± 14.3	84.3 ± 29.2	84.3 ± 29.2	0.115
Progesterone (nmol/L)	0.38 ± 0.2	0.43 ± 0.2	0.41 ± 0.1	0.327
**Perceptual Scores**				
*Pain*	*4.2 ± 2.1*	3.5 ± 2.8	*2.9 ± 2.4*	0.541
Mood	3.2 ± 1.7	2.8 ± 2.6	3.5 ± 1.6	0.834
*Soreness*	*1.2 ± 0.8*	*2.3 ± 2.6*	*2.6 ± 1.9*	0.288
General Fatigue	3.6 ± 2.6	2.8 ± 1.9	3.1 ± 1.1	0.643
*POMS Energy* *Index*	*1.6 ± 5.4*	*5.6 ± 5.6*	*2.9 ± 5.2*	0.125
**Bilateral Hops**				
Pavg (W/kg)	27.0 ± 5.2	28.7 ± 3.7	28.5 ± 4.9	0.382
Tcont (ms)	224.5 ± 13.0	223.7 ± 27.5	222.1 ± 25.5	0.966
Tflight (ms)	358.9 ± 51.4	381.0 ± 34.6	361.8 ± 43.2	0.218
Ratio (Tflight/Tcont)	1.58 ± 0.29	1.70 ± 0.30	1.61 ± 0.32	0.491
**Counter movement jump**				
Pavg (W/kg)	19.6 ± 3.4	18.5 ± 2.9	18.9 ± 3.1	0.213
**Tflight (ms)**	**421.8 ± 27.9**	**421.0 ± 35.6 ** ^#^	**408.8 ± 31.8**	0.033 *
		**Handgrip**		
Right (kg)	35.6 ± 5.3	37.0 ± 4.9	35.6 ± 4.9	0.144
Left (kg)	33.8 ± 6.3	34.4 ± 6.8	34.0 ± 6.0	0.628
**Isometric Strength**				
Knee Extension (Nm)	153.7 ± 40.1	152.7 ± 41.6	157.9 ± 38.6	0.617
**Isokinetic Torque**				
60° s^−1^ Extension (Nm)	105.1 ± 32.4	104.5 ± 30.5	110.1 ± 27.9	0.569
60° s^−1^ Flexion (Nm)	75.9 ± 16.7	76.8 ± 14.1	78.9 ± 19.6	0.708
*240° s^−1^ Extension (Nm)*	*61.4 ± 12.3*	62.5 ± 13.0	*67.0 ± 9.5*	0.291
240° s^−1^ Flexion (Nm)	56.5 ± 13.0	57.8 ± 9.3	61.9 ± 12.6	0.071

Data shown as mean ± SD. * **Bold** Significant change over the cycle (*p* < 0.05). ^#^ Post hoc test significantly different from late hormone phase. *Italic* Moderate or large effect size between phases. Pavg: average power, Tcont: contact time, Tflight: flight time.

## Data Availability

The data that support the findings of this study are available on request from the corresponding author. The data are not publicly available due to privacy or ethical restrictions.

## Supplementary Materials

The following are available online at https://www.mdpi.com/article/10.3390/ijerph182010565/s1, Supplementary File: Combined Monophasic Oral Contraceptive Formulations.
