# Supplementary material for: The Effect of the Menstrual Cycle and Oral Contraceptive Cycle on Muscle Performance and Perceptual Measures
Source: Int J Environ Res Public Health. 2021 Oct 9;18(20):10565. doi: 10.3390/ijerph182010565 (PMC8536049; doi:10.3390/ijerph182010565)
Supplement: Supplementary file 1 [file ijerph-18-10565-s001.zip › ijerph-1384709-supplementary.pdf]

Supplementary File: Combined Monophasic Oral Contraceptive Formulations

Trade Name	Number of Participants	Ethinyl Estradiol Dose (mg)	Progestin Type	Progestin Dose (mg)	Androgenicity
Brenda® 35 ED	3	0.035	Cyproterone	2.00	NA[#]
Brevinor®	1	0.035	Norethindrone	0.50	0.5
Diane® 35 ED	1	0.035	Cyproterone	2.00	NA[#]
Levlen® ED	7	0.030	Levonorgestrel	0.15	1.25[*]
Micronelle® 30 ED	2	0.030	Levonorgestrel	0.15	1.25[*]
Monofeme®	1	0.030	Levonorgestrel	0.15	1.25[*]
Norimin-1®	2	0.035	Norethindrone	0.50	0.5
Reclipsen®	1	0.030	Desogestrel	0.15	0.51

Progestin potency and androgenicity calculated by method of Greer et al. (2005)

* OC with high androgenicity (≥1.0)

[#] Anti-androgen so no androgenicity values, classified in low androgenicity group
